# Dental Restorations

**DOI:** 10.3390/bioengineering10070820

**Published:** 2023-07-10

**Authors:** Mauro Mandurino, Giovanna Laura Di Domenico, Sofia Baldani, Giacomo Collivasone, Enrico Felice Gherlone, Giuseppe Cantatore, Gaetano Paolone

**Affiliations:** Dental School, IRCCS San Raffaele Hospital, Vita-Salute University, 20132 Milan, Italygiovannalauradd@gmail.com (G.L.D.D.); s.baldani@studenti.unisr.it (S.B.); g.collivasone@gmail.com (G.C.); gherlone.enrico@hsr.it (E.F.G.); cantatore.giuseppe@hsr.it (G.C.)

## 1. Novelties in Restorative Dentistry and Endodontics

Fulfilling a patient’s request for a healthy, functional and esthetic smile represents a daily challenge for dental practitioners. A rise in requests for esthetic dental treatments has led to the introduction of new materials, treatments and procedures able to provide non-invasive, long lasting and predictable treatment options [1,2,3]. The aim of this article is to present the latest innovations concerning the dental field reported by this journal.

This collection of articles related to restorative dentistry and endodontics [4,5,6,7,8,9], published in *Bioengineering*, includes novel restorative solutions for the most common problems that clinicians and patients can relate to: dental decay and dental fracture [10,11]. Regarding dental fracture, traumatic dental injuries (TDIs) concern mostly children and young adults [12]. In most cases, dental injuries involve anterior teeth, especially the maxillary upper incisors. Crown fractures, with or without pulp exposure, are the most common type of trauma in permanent dentition [13]. Radwanski et al. [4] aimed to present a possible conservative treatment for complicated tooth fracture in a case report. This technique consisted of partial pulpotomy followed by the adhesive reattachment of the tooth fragment using a pre-heated resin composite [4]. When a tooth fragment is available and viable for the clinicians to work on it, it should be reattached [14]. To achieve the best level of structural strength and aesthetic mimicry of the reattached fragment, clinicians should instruct patients (and usually the children’s parents) on how to store, retrieve, and rehydrate the fragment. In this clinical case, after the application of MTA on one of the two upper incisors during a complicated pulpo-dentinal fracture, the fragment was reattached without any modifications sustained to the tooth or the fragment. In both teeth and fragments, the selective enamel etching was performed using a 36% phosphoric acid gel under rubber-dam isolation. Then, a self-etching two-component adhesive system was applied following the manufacturer’s instructions, and air-dried with a strong stream of air for 5 s to completely remove the excess adhesive before light curing. Subsequently, a thin layer of enamel composite was applied directly on the tooth as an intermediate material to perform fragment reattachment. The composite was previously heated up to 54 °C to allow the better adaptation of the fragment to the tooth and to provide more manageable removal of the excess resin [15]. The adhesive reattachment of a fragment, combined with vital pulp-therapy procedures, represents the first-choice treatment option in cases of both simple and complicated crown fractures [4,16]. This technique may therefore help the clinician and allow a better outcome.

The other major problem that dentists deal with daily is the treatment of tooth decay. Dental caries has been classified as the most common oral disease, as it affects around 60–90% of adolescents and 100% of grownups around the world [17,18]. The progression of dental decay and the involvement of the pulp in the carious lesion lead firstly to clinical scenarios of pulpitis and then eventually to necrosis of the pulp. It is simple to understand how untreated or undiagnosed caries could lead to endodontic treatments, and if not carried out correctly, to the loss of the tooth. Clinicians and patients should therefore always agree on intervening as soon as possible when carious lesions are diagnosed. Moreover, the best material and technique should be prioritized in order to prevent secondary caries. Currently, dental restorations performed with adhesives and composites represent the state of the art. However, the inherent composition of dental adhesives and composites has a high affinity for dental plaque (biofilms) accumulation, leading to secondary caries [19,20,21,22,23]. Additionally, resin-based restorative materials are highly susceptible to degradation, which may compromise the duration of such restorations over time [24,25,26,27,28,29,30,31,32]. The deterioration of the restoration margin is strictly correlated with that of the hybrid layer [33]. The hybrid layer is the interface created by the application of the adhesive system; in this thin layer elution is one of the major causes of void creation. The presence of these voids enhances the proteolytic activity of endogenous matrix metalloproteinases (MMPs) which leads to secondary caries progression. As explained by Beck and Ilie [6], different studies were produced to develop simplified, clinically applicable and efficient protocols for the usage of photoactivated Riboflavin (RB) in the dentin bonding process. Riboflavin—probably better known as vitamin B2—is a compound that, thanks to its activity, could reinforce the collagen network by cross-linkage, making it more resistant to proteolytic attacks. Their in vitro study published in *Bioengineering* aimed to test a primer incorporated with RB 3% (wt/vol) [6]. Their results, on extracted human teeth, showed that even though some parameters might indicate less long-term degradation for RB test groups, the lower shear bond strength (SBS) values show that RB-photosensitized crosslinking cannot be transferred to clinical applications yet. On the other hand, researchers and industries are continuously developing RBCs and adhesive systems characterized by prolonged antibacterial agent release or contact-killing surfaces [34,35]. Antimicrobial nanoparticles may, for example, be of particular value [36]. The potential ability of nanoparticles to control oral biofilms is gaining popularity. Thus, their physicochemical characteristics, such as their degree of hydrophobicity, surface charge, and the surface-area-to-mass ratio of the plaque biofilm, are being investigated. Incorporating antibacterial compounds inside an RBC seems to reduce the risk of failure due to secondary caries. Moreover, nanoparticles may act in different ways: by causing cell lysis due to their interaction with the peptidoglycan cell wall and membrane; by altering protein synthesis; or by preventing DNA replication. Nevertheless, as reported by Balhaddad et al. [7], although the current data seem promising, long term results are lacking, resulting in an uncertainty on their reliability after aging. Moreover, potential cytotoxicity may be hypothesized and studies investigating it in the literature is scarce.

As previously mentioned, the lack of diagnosis and treatment of carious lesions leads to evolution of various presentations of pulp pathology which results in the endodontic treatment of the tooth. The purpose of root canal treatment (RCT) is to maintain the function of a tooth, cure disorders of the pulp, and prevent and treat diseases of periapical tissue. Although any RCT performed correctly, in compliance with the guidelines, can give excellent results over time, there is no doubt that the operator’s experience can play a fundamental role in the success of the therapy itself. In the study carried out by Pietrzycka et al. [8] different groups of students (4th and 5th grade) and operators with different levels of experience were compared to understand if there were some differences in the quality of RCTs and in the number of visits needed to complete a primary RCT. To compare clinical results, the post-endo rx was analyzed according to several parameters, like the length and density of the RCT, and was linked with different filling criteria (adequate, overfilling, short filling or inadequate). The results showed that both the largest number of visits and the lowest quality of RCT was performed by students in the 4th grade. In contrast, endodontists needed the lowest number of visits to complete RCT and reached a higher quality of root canal filling compared to the other operator groups. Nevertheless, a major limitation of this study was that different groups used different types of instruments (manual or rotary) to complete the RCT [8]. After a successful endodontic treatment, which includes a proper access cavity, shaping, irrigation, and the tri-dimensional filling of the root canal space, clinicians need to select the most appropriate restoration for each endodontically treated tooth (ETT) [37,38,39,40]. Very often, it is not easy to restore endodontically treated teeth, due to the lack of residual dental structure [32,41]. Over time we have moved from metallic-post-type restorations, which were cemented traditionally inside the canal, to restorations that are based on the placement of fiber-posts, which have the advantage of being cemented following adhesive procedures [42]. However, it has been demonstrated and debated in the literature that post-placement leads to the excessive removal of the dentin substance [43]. For this reason, industries are evolving towards the use of multi-fiber-reinforced composite (mFRC) based on a bundle of fibers that are bonded directly to the root canal. The application of mFRC into the root canal space does not require any use of post-space preparation [44]. Therefore, the adaptation of mFRC to the root canal anatomy, without additional dentin removal, may be of advantage for tissue preservation [45]. Kharouf et al. [5] investigated the bond strength to root canal dentin and the filling ability of a new multi-fiber-reinforced composite post (mFRC) compared to a conventional single-fiber-reinforced composite post (sFRC). In this in vitro study, extracted human premolars were firstly selected, scanned with a CBCT and instrumented by the same operator, then the sFRC and mFRC were placed into the canals after adhesive treatment was carried out. The adaptation and the presence of voids was analyzed by using scanning electron microscopy (SEM). Moreover, the adhesion of the two types of composites was studied via the push-out bond strength (PBS). The mFRC exhibited superior results in filling ability compared to sFRC. However, the bonding ability of the mFRC to dentin may have been lower at the coronal third compared to the sFRC [5]. 

Continuing in the field of endodontics, one of the challenges that has required the commitment of clinicians and researchers in recent decades is the controversial topic of regenerative endodontics. In necrotic teeth with immature roots, the possibility of restoring their full functionality is an attractive goal, since the regeneration of lost tissues improves the longevity of these elements. This topic is discussed in a review published in *Bioengineering* [9], which firstly explains the various differences between the state-of-the-art regenerative endodontic techniques and then introduces their new regenerative approach, in order to restore the homeostatic balance of the treated tooth. The use of multipotent-stem-cell (MSC)-derived secretome, such as CM or EV, instead of cell therapy, can promote the stimulation of proliferation and differentiation of MSC from dental pulp towards odontoblasts, blood vessels and nerves. This concept may represent the basis for a new regenerative endodontics strategy.

## 2. Novelties in Implantology

When tooth preservation is not possible, tooth extraction appears inevitable. Once a tooth is lost, rehabilitation may require the placement of an implant. The dimensional bone and soft-tissue alterations following tooth extraction have a significant impact on the esthetic outcome of implant-supported restorations [46]. Several preclinical and clinical studies showed that, during the first weeks following a tooth extraction, remodeling activity takes place, resulting in marked bone resorption, mainly at the level of the buccal wall [47,48,49].

Over recent decades, several surgical procedures, denominated “alveolar ridge preservation”, have been introduced, aiming to maintain the existing soft and hard tissues as well as a stable ridge volume, to simplify subsequent treatment procedures and to optimize functional and aesthetic outcomes [50]. Currently, a broad spectrum of grafting materials of different origins, such as allografts, xenografts, and alloplastic materials, are available [51,52]. A recent randomized controlled clinical trial evaluated the effect of a solid PRF on the soft-tissue healing of molar and premolar extraction sockets at 3 months follow-up [53]. When the test group (PRF-treated) was compared to a control one (non-PRF-treated), results demonstrated that PRF leads to a significantly faster ridge sealing and a lower contraction of the wound. Contrarily, no differences between the two groups in terms of patients’ pain perception were reported. However, when bone dehiscence is present at the time of implant placement, different bone regenerative techniques, such as guided bone regeneration (GBR), need to be used to allow the correct placement of a dental implant. GBR is a successful, well-documented and widely used dental surgical procedure to treat various alveolar bone defects, using membranes and bone-grafting. It allows the surgical site to be colonized by bone-tissue cells surrounding the regeneration area and the re-establishment of local bone volume [54,55]. Moreover, it can be performed before or at the time of implant placement. Different protocols and a combination of biomaterials have been proposed for bone-regeneration procedures. A recent pre-clinical study was conducted in order to evaluate a new technology for producing a cell-seeded fibrin gel with the same shape and size of the bone defect to be treated [56]. The scaffold was composed of fibrin glue and dental-pulp stem cells (DPSC) coming from the periodontal ligament, that were layered onto the 3D-printed scaffold surface. The authors concluded that the combination of DPSCs and fibrin gel can be used to promote bone regeneration. Indeed, the fibrin glue is a sufficient material for scaffolds with good mechanical characteristics, while the DPSCs maintain their viability, immunophenotype and osteogenic potential. Funato et al. [57] described, in a retrospective case series, a minimally invasive novel resorbable membrane-pouch technique, performed in conjunction with implant placement, where collagen membranes were secured to the periosteum without the need of titanium pins. A total of 11 patients were included and treated with immediate (*n* = 3) or delayed implants (*n* = 8). The surgical procedure consisted of a full-thickness flap elevation, which was connected to a partial-thickness flap in the basal area of the maxilla, and of a prosthetically driven crestal-implant placement. Then, a resorbable membrane was trimmed to fit and was inserted beneath the periosteum and secured to it using resorbable sutures. Demineralized bovine bone mineral was positioned in the internal space of the pouch, surrounded by the resorbable membrane and exposed labial implant surface. Finally, to achieve good esthetic soft-tissue results, a connective-tissue graft (CTG) was also placed on the buccal superior aspect and secured to the periosteum or mucosal flap. Results showed that all implants were successful and functional, with optimal soft-tissue health and esthetics. A key element for the high aesthetic score seems to be the simultaneous CTGs performed. Indeed, in recent years, soft-tissue management with the use of connective tissue grafts around implants is of utmost importance to mimic natural ideal conditions, and for this reason it has become a topic of growing interest for clinicians. Several studies investigating the augmentation of buccal soft tissue with autogenous CTG in conjunction with an IIP have showed reliable results compensating for the volume changes of the facial tissues after implant placement [58,59]. Among these, a recent study indicated that the addition of a CTG at the time of an IIP seems to reduce the horizontal changes of the alveolar ridge that occur, and allows the maintenance of the tissue contour due to an increase in soft-tissue thickness [60]. 

Although implant therapy has undergone significant improvements, complications are still reported with a high frequency. There are two categories of complications that occur in implant therapy: biological and technical (mechanical) [61]. 

“Biological complications” refer to disturbances in the function of the implant, characterized by biological processes that affect the supporting tissues, including peri-implant diseases [62,63]. Etiological and risk factors for peri-implant diseases have been identified in several experimental and clinical studies performed in recent years [62,64,65]. Microbial plaque accumulation is considered to be the most important factor in the pathogenesis of peri-implant diseases, similarly to periodontitis, and the presence of micro-gaps at the implant–abutment interface has been implicated in several studies as the source of bacterial penetration [66]. This bacterial reservoir can negatively impact the health of the peri-implant tissue, leading to inflammation and bone loss. A recent in vitro study evaluated the microleakage of two bacterial species with different diameters (*S. oralis* and *P. aeruginosa*) at the implant–abutment interfaces of three different implant connections (external hexagon, internal hexagon, cone morse taper internal connection) [67]. Results indicated that although the components were assembled according to the manufacturer’s recommendations, microleakage of the selected microorganisms occurred. Typically, bone resorption in peri-implantitis lesions affected the more coronal region of the implant and only gradually proceeded in the apical direction [68]. Occasionally, peri-implantitis may develop apically, in the same way that a dental periapical lesion does (without the coronal crest bone being involved). This type of lesion is indicated with the term “implant periapical lesion” and it represents a distinct pathological entity [69]. Luongo et al. [70] described two different approaches to treat this type of peri-implant periapical lesion: the removal of the implant (case n.1) or the removal of its apical portion (case n.2). According to the authors, the last treatment option appeared to be predictable, since it allowed elimination of the inflammatory tissue and the maintenance of the remaining part of the fixture, which became optimally osseointegrated. 

“Technical complications” serve as a collective term for mechanical damage to the implant/implant components and supra-structures. One of the key elements impacting osseointegration is the stability of the connection between the fixture and the abutment. In particular, it may be difficult and complicated to achieve a passive fit when restoring several, non-parallel, and splinted implants. In these clinical situations, non-engaging abutments are one of the several types of abutment connections that are available and are frequently utilized in multi-unit cases where they need to have a passive fit and not exert too much stress on the implants [71]. However, limited evidence is available on the stress distribution of these type of abutments. For this reason, using finite element analysis, a recent study aimed to evaluate the biomechanical characteristics of a new EZ-post non-engaging abutment system of a BlueDiamond^®^ implant (BD group; Megagen, Bedfordshire, UK), compared to a similar system of an AnyOne implant (AO group; Megagen, Bedfordshire, UK) [72]. On the basis of the obtained results, the authors concluded that the new EZ-post non-engaging abutment of the BD implant may be used in clinical settings in a manner similar to that of the AO abutment system. Indeed, results showed that the fixture and the stress distribution, at the level of surface where the fixture and the abutment make contact, were lower in the AO group than in the BD group. Nevertheless, at the same time, the AO group had a greater incidence of abutment fracture, compared to the BD group. In order to obtain properly osseointegrated implants, new surface treatments are being introduced as well as new handpieces. Aysesek et al. [73] investigated, in their study, the topographical, chemical and osseointegration characteristics of an implant surface treated with sandblasting and acid etching, compared to a new boron and boric-acid (H3BO3) treatment in sheep tibia. They concluded that boron treatment and coating produced a smoother surface compared to the conventional one. This property seemed to cause lesser resistance against reverse rotational forces in the short term (3 weeks). Nevertheless, in the long term (7 weeks), it provided significant resistance to rotational forces and no adverse reactions. Boric acid treatment seems to be promising and deserves further investigations to fully comprehend which is the best dose and method of application.

On the other hand, Park et al. [74] compared the osseointegration of dental implants placed using two dental laser-implant handpiece systems. Implants were placed in rabbit tibia using a conventional laser-implant handpiece and a multi-laser handpiece system under development. They found no statistically significant difference between the two groups. Therefore, considering the limitations of this study, the type of laser-implant handpiece did not seem to affect osseointegration and soft tissue healing. For many years, an alternative to dental implants has been sought, and recently Bektas et al. [75] described the characterization of tooth-germ organoids cultured with hydrogel microparticles. These have recently demonstrated their role in tissue repair and regeneration, facilitated by their characteristics. Furthermore, they possess a scaffold ability, the effective cell and drug delivery, and a property to mimic the extracellular matrix. This material served as an extensive surface area for human dental-pulp stem cells and porcine dental epithelial cells to attach and proliferate to. Interestingly, it self-assembled into organoids, within which the cells managed to maintain their viability and morphology during the incubation period. These organoids reached a volume of ~50 mm^3^ within two weeks of culture. Both the human-pulp stem cells and the porcine epithelial cells attached to each other without any external intervention. These findings demonstrate the tooth-regeneration potential of tooth-bud organoid-like structures. 

## 3. Novelties in Orthodontics

Orthodontics is a pivotal branch in dentistry, which allows tooth movement through different devices. Although fixed orthodontic treatments demonstrate notable advantages [76,77], one of their widely reported side effects is oral mucosa lesions due to the presence of sharp edges [78,79,80]. Traumatic oral ulceration (TOU) occurs during the first period of treatment and may cause pain during feeding or speaking, compromising patients’ compliance [78]. TOUs disappear within 10–14 days after removing the origin cause [81]; meanwhile, it could be useful to self-medicate the injury [82] or apply orthodontic wax on the braces to relieve pain [83]. Among tested products that aim to prevent TOUs or to speed up healing [84,85], a new gel (BMG0722) (BMG Pharma S.p.a., Milan, Italy) has been evaluated in Tremolati’s study [86]. It is composed of hyaluronic acid (HA) and polyvinylpyrrolidone (PVP): PVP creates a barrier that allows both a decrease in pain and faster healing [85], while HA reduces inflammation [87,88] and promotes the healing process, as it is also used in other dentistry treatments, for example, after scaling in chronic periodontitis [89,90]. In the literature, its effectiveness regarding TOU treatment has been proved [91]; furthermore, Tremolati et al. [86] analyzed the different results on the healing process of TOUs between the combination of orthodontic wax and BMG0722 gel, and the combination of orthodontic wax with a placebo gel, during orthodontic treatment. In this double-blind RCT, a VAS-scale test and lesion measurements at T0, T1, and T2 were examined: out of 110 subjects screened, only 57 patients terminated the follow-up period. Thus, this study confirmed that this new combination speeds up healing and reduces pain symptoms (VAS score at 5 days after TOU onset) compared to the orthodontic wax and placebo combination (VAS of 0.44 and 4.00, respectively), and also diminishes the dimension of a lesion after 5 days of treatment [86]. Thanks to its ability to reduce oral mucosa pain [92] and enamel lesions due its conventional bracket system [93], to enable the treatment of severe orthodontic disorders [94] and to allow better comfort, esthetics and hygiene, orthodontic treatment using clear aligners has gained popularity. Through the growth of producer companies [95], nowadays, over 10 million patients have tried out this new orthodontic technique [96]. Nevertheless, the main side effect is that, although general treatment time has decreased compared to conventional systems, in extraction cases it has increased.

Clear aligner treatment (CAT) has developed thanks to software planning, improved transparent thermoplastic materials and resin attachments [97,98]. Although they are often composed by cytotoxic materials, such as polymethyl methacrylate (PMMA), the curing process might decrease this potential risk, but not avoid it totally [95]. Indeed, incomplete conversion seems to increase the release of monomers, such as methyl methacrylate (MMA), triethylene glycol dimethacrylate (TEGDMA), 2-hydroxyethyl methacrylate (HEMA), and bisphenol A glycidyl methacrylate (Bis-GMA) [99], that could cause cytotoxicity, mutagenicity, teratogenicity and estrogenicity [100]. Thus, Francisco’s systematic review aimed to analyze the release of toxic monomers from 3D resin devices and their systemic influences, and included in vivo and in vitro studies [101,102]. A total of 14 articles were evaluated, but the risk of bias was considered medium/high; hence, the results of the monomers released by resin are not reliable and the literature needs more studies with the same methods, aligner fabrication and resin components to reach more comparable results [103]. Meanwhile, the orthodontists must pay attention to apply the aligners only for short treatments, with patients at non-fertile ages, and instruct them to take care with it, especially through avoiding hot foods [101]. As previously mentioned, CAT could also treat complex malocclusions thanks to the forces exerted on teeth [104]. Multiple studies have followed the development of new techniques to measure orthodontic forces [99,105], but a universal device capable of precisely detecting the strength applied by orthodontic treatments has not yet been found. For this reason, Lee’s study [96] shows an innovative orthodontic force-measurement method which allows the monitoring of the deformation of a pliant semi-sphere sensor, composed of a biocompatible polymer bonded to the inner aligner surface or to the tooth surface. The sensor is economic, simple and states the symmetry and homogeneity of the forces on the attachment, calculating the exerted force and the resulting orthodontic movement over the tooth’s surface. The change in the contact area in the semi-sphere sensor was measured by a microscope, while the changing force can be monitored by changes to the contact area between the sensor and the aligner’s inner surface. Hence, the sensor could establish the force exerted on a specific sensor part: further improvements to this 3D sensor would be a group of numerous semi-sphere sensors in different positions for the directional investigation of the forces [96]. Furthermore, it could also be useful to analyze forces applied in more complex situations, such as in malformations or transversal deficits. Vale et al. [106] investigated mandibular hypoplasia treatment; this is the most common growth disorder of the facial skeleton in Western Europe, ranging from 41% to 56%. It could be related to congenital malformation syndromes, angle class II occlusions, convex profile, and mandibular deficiency [107]; the need of orthodontic-surgical treatment [108] to correct dental occlusion, masticatory and respiratory function in cases of class II skeletal malformations [109] must be evaluated in relation to their severity. Indeed, although the orthodontic–surgical–orthognathic technique is widely diffused [110], it has major risks and a long treatment time (it is divided into four stages: the presurgical orthodontic phase, the surgical phase (BSSO), the post-surgical stage and an optional surgical-intervention stage to eliminate the osteosynthesis plates) and it is only suggested for adult patients, since skeletal growth is finished. For previously mentioned reasons, other procedures, such as mandibular distraction osteogenesis (DO), for dentofacial-deformity treatment have been investigated [111]. In the literature, results were not homogeneous: several systematic reviews assessed that both BSSO and DO methods can lead to enhanced hard and soft tissue, but the DO technique has less risk of relapse after five years follow-up [112]. Since external distractors have several side effects (discomfort, psychosocial problems because of the distractor’s perceptibility and dimension, instability in the anchorage, the risk of dental and/or nerve lesion, and the presence of edema or localized infections), intraoral distractors have mainly been used to treat transverse jaw deformities, causing less psychosocial disorders thanks to their diminished volume, lower infection rate without the need of surgery, lower morbidity and better stability in fixation [113]. Nevertheless, difficulties in managing the vector direction to avoid asymmetry or posterior mandible rotation persist [114]. Recently, a new intraoral individual distractor for the lower jaw, bonded in the first molar and lower canine, was tested by Vale’s study: it included a stainless-steel disjunction screw that, through rotation-pressure, enables expansion up to 12 mm [106]. When applying this new device in a distraction osteogenesis technique, soft tissues better adapt to the gradual bone elongation compared to in the BSSO technique, decreasing the relapse rate arising from robust soft-tissue tensions [115]. Hence, distractors should be evaluated for the treatment of lower jaw hypoplasia and retrognathia, instead of conventional surgical treatment, for their improvements regarding the patient’s toleration and their development of the anterior mandibular bone segment, without posterior mandibular rotation or an influence on the gonial angle or transverse angulation of the segments [106].

As previous mentioned, transversal deficit is another worldwide disorder usually associated with malocclusions, often those of the upper-jaw-present single or bilateral cross-bite [116]; predisposing factors are congenital alterations, dystrophies, metabolic disorders, infections, trauma, atypical swallowing or oral breathing [117]. Skeletal and dental expansion are the primary treatment for a skeletal or dentoalveolar transverse deficiency [118]. Expansion treatment lasts two or four months, in addition to a retention period (from 4 to 6 months), but it could be faster during childhood thanks to the fibrous texture of the median palatine suture [117]. Although several orthodontists solve this malocclusion with TADs (Temporary Anchorage Devices), many colleagues also achieve excellent results with rapid or slow expander: rapid palatal expander in deciduous or early mixed dentition, or slow expander (NPE-2 or similar) in permanent dentition. The rapid palatal expander (RPE) performs fast expansion, activating considerable forces at the sutural site by using a key [119]. Through this fixed device, it was demonstrated that after two weeks, there is a rise in width due to skeletal growth of 80% and due to teeth’s movement of 20%, while in patients treated with slow expander, like Nitanium^®^ Palatal Expander™, the rise in width is 50% orthodontic and 50% orthopedic. Furthermore, rapid palatal expander (RPE) and Nitanium Palatal Expander-2 (NPE-2) were compared in Montaruli’s [120] retrospective study to assess their upper-jaw expansion effectiveness on thirty-six subjects with a mild to severe transverse maxillary deficiency and unilateral or bilateral posterior cross-bite. After a digital analysis of 3D models, the measurements of the anterior arch width, the posterior arch width, the palate height, and palatal surface were investigated and all data collected in this study indicated that both devices were suitable for solving transverse deficiency, with the choice of the most suitable related to patients’ age and the type of dentition [120].
